# Network of Interactions Between Ciliates and Phytoplankton During Spring

**DOI:** 10.3389/fmicb.2015.01289

**Published:** 2015-11-20

**Authors:** Thomas Posch, Bettina Eugster, Francesco Pomati, Jakob Pernthaler, Gianna Pitsch, Ester M. Eckert

**Affiliations:** ^1^Limnological Station, Institute of Plant Biology and Microbiology, University of ZurichKilchberg, Switzerland; ^2^Department Aquatic Ecology, Swiss Federal Institute of Aquatic Science and TechnologyDübendorf, Switzerland; ^3^Microbial Ecology Group, Consiglio Nazionale Delle Ricerche- Istituto per lo studio degli ecosistemiVerbania Pallanza, Italy

**Keywords:** ciliate morphotypes, ciliophora, local similarity analysis, phytoplankton spring bloom, network analysis

## Abstract

The annually recurrent spring phytoplankton blooms in freshwater lakes initiate pronounced successions of planktonic ciliate species. Although there is considerable knowledge on the taxonomic diversity of these ciliates, their species-specific interactions with other microorganisms are still not well understood. Here we present the succession patterns of 20 morphotypes of ciliates during spring in Lake Zurich, Switzerland, and we relate their abundances to phytoplankton genera, flagellates, heterotrophic bacteria, and abiotic parameters. Interspecific relationships were analyzed by contemporaneous correlations and time-lagged co-occurrence and visualized as association networks. The contemporaneous network pointed to the pivotal role of distinct ciliate species (e.g., *Balanion planctonicum, Rimostrombidium humile*) as primary consumers of cryptomonads, revealed a clear overclustering of mixotrophic/omnivorous species, and highlighted the role of *Halteria*/*Pelagohalteria* as important bacterivores. By contrast, time-lagged statistical approaches (like local similarity analyses, LSA) proved to be inadequate for the evaluation of high-frequency sampling data. LSA led to a conspicuous inflation of significant associations, making it difficult to establish ecologically plausible interactions between ciliates and other microorganisms. Nevertheless, if adequate statistical procedures are selected, association networks can be powerful tools to formulate testable hypotheses about the autecology of only recently described ciliate species.

## Introduction

In the original description of the PEG-model (Plankton Ecology Group, Sommer et al., 1986) explaining the mechanisms of plankton successions in lakes, the authors state about phytoplankton spring bloom dynamics: “*It is clearly to be seen in most of the lakes that the first herbivores to build up abundant populations in the spring are small protozoans and rotifers, which have short generation times and exponential increase within a few days*.” Although the role of microzooplankton groups as the first relevant grazers of algal spring blooms was highlighted by the PEG-model authors, this trophic link has been almost forgotten -or overlooked- for decades. Conversely, several studies have focused on a direct trophic shortcut from phytoplankton to metazooplankton (e.g., daphnids, copepods), attributing to the latter the sole control of algal development during spring. Already in the early 1990s, Helga Müller and co-workers published a pioneering work on the importance of ciliates (Ciliophora) as the first and most effective grazers of phytoplankton spring blooms in Lake Constance (Müller, 1989; Müller et al., 1991a; Müller and Weisse, 1994). These observations have also been confirmed for other temperate lakes (Amblard et al., 1993; Sommaruga and Psenner, 1993; Mathes and Arndt, 1995; Carrias et al., 1998), and the role of protists as consumers was highlighted in a recent description of the PEG-model (Sommer et al., 2012). Furthermore, it was recognized that the spring peak of algivorous ciliates is followed by a conspicuous succession of various mixotrophic (Amblard et al., 1993), omnivorous and predatory ciliate species (Müller et al., 1991b). Due to their fast generation times (hours to days), the succession of ciliate species is characterized by several short-lived peaks of a few dominant genera and a high sampling frequency is thus required to follow their dynamics in “real-time” (Šimek et al., 2014).

Nevertheless, there is an obvious discrepancy between the considerable knowledge on the diversity of freshwater ciliate morphotypes (summarized in Foissner et al., 1999), their succession during spring (Weisse and Müller, 1998; Sonntag et al., 2006; Zingel and Nõges, 2010), and the scarce information on species specific interactions between ciliates and other microorganisms (protists and bacteria). In order to determine these factors, there is a need for broader studies on microbial food webs that examine multiple abiotic parameters in parallel with micro-organisms at high taxonomic resolution. Simultaneous information of the diversity of organisms and a detailed pattern of their co-occurrences can be obtained via next generation sequencing of phylogenetic marker genes and software-based network analysis (Steele et al., 2011; Chow et al., 2014). However, in the case of freshwater ciliate species, this approach has several limitations. (i) Sequence information is still missing for many well-known and precisely described freshwater ciliate morphotypes (Stoeck et al., 2014). It is therefore difficult to relate operational taxonomic units (OTU) with the existing knowledge about the autecology of morphospecies (see literature reviews in Foissner et al., 1999; Lynn, 2008). (ii) The co-occurrence pattern of a ciliate OTU with, e.g., algal OTUs, does not inform about the type of interaction between them at all, when no autecological background information is consulted. (iii) Due to the high copy number of 18S rRNA (ribosomal ribonucleic acid) genes in single ciliate cells (Gong et al., 2013), a reliable quantification of ciliate abundance is not yet possible solely on molecular techniques.

As a consequence, the identification and quantification of ciliates based on their morphology might currently be a more direct means to investigate interspecific interactions. Here we present data from a high frequency sampling campaign (2–4 day sampling intervals during 7 weeks) aimed at characterizing the dynamics of ciliate morphotypes during a phytoplankton spring bloom in a large freshwater lake (Lake Zurich, Switzerland). A contemporaneous statistical analysis was conducted using the abundances data of protistan morphotypes. We searched for interspecific associations, keeping in mind autecological background information for the detected morphospecies. We also tested the explanatory power of an ecological network based on local similarity analysis (LSA), a method often used for the evaluation of environmental sequences data allowing for the detection of time-shifted co-occurrences between parameters.

## Methods

### Study area and sampling site

Lake Zurich is an oligo-mesotrophic pre-alpine lake with a maximum depth of 136 m and a surface area of 66.8 km^2^. The entire water volume of 3.34 km^3^ is theoretically renewed in 1.2 years. The lake is located in a densely populated area and serves as a source of drinking water for more than 1 million people. Lake Zurich is monomictic with infrequent holomixis (Posch et al., 2012). Since the beginning of the 20th century the trophic status has increased and eutrophication reached its maximum in the 1960′s. Due to consequent waste water treatment total phosphorus-concentrations have decreased from >120 μg L^−1^ to presently about 15 μg L^−1^.

### Sampling strategy

**Figure 1:**
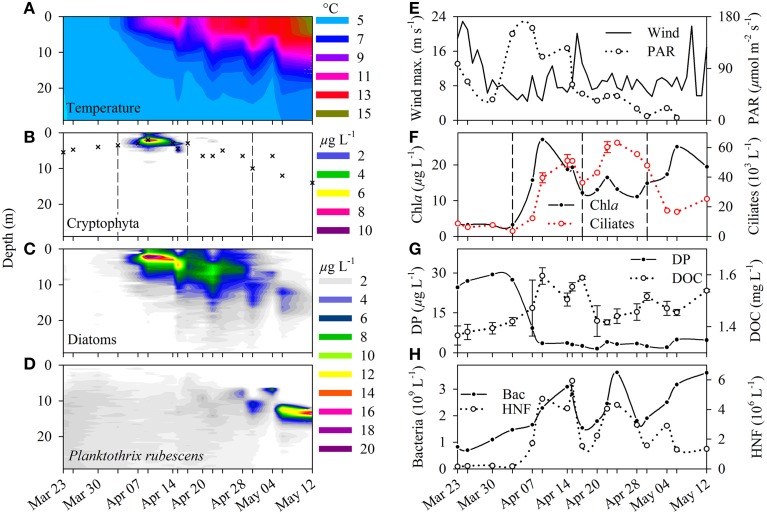
**(A)** Water temperature, **(B)** cryptophytes-related Chlorophyll *a* (Chl *a* in μg L^−1^), **(C)** diatom-related Chl *a* (μg L^−1^), **(D)** *Planktothrix rubescens* related Chl *a* (μg L^−1^) of the surface water body (0–30 m) in Lake Zurich during a phytoplankton spring bloom (March–May 2009). Crosses in **(B)** indicate the sampling depth, and dashes in **(B,F)** indicate the four phases of the investigation period (see text). **(E)** Maximal wind speed (m s^−1^) at the surface of the lake and photosynthetic active radiation (μ mol quanta m^−2^ s^−1^) measured in the sampling depth. **(F)** Total Chl *a* (μg L^−1^) and ciliate abundance (10^3^ L^−1^). On three selected sampling dates we made triplicate QPS preparations to evaluate standard deviations of ciliate counts. **(G)** Concentration of dissolved phosphorus (DP, μg L^−1^) and dissolved organic carbon (DOC, mg L^−1^, average ± standard deviation). **(H)** Bacterial (10^9^ L^−1^) and heterotrophic nanoflagellate (HNF, 10^6^ L^−1^) abundance in the sampling depths during the investigation period.

Samples were taken in 2–4 day intervals at one sampling site (47°19.3′N 8°33.9′E, z_m_ = 100 m) from 23 March to 12 May 2009 at around 10 a.m. In spring, the weakly stratified water body of Lake Zurich is very susceptible to changes in weather conditions and especially to storm events (Bleiker and Schanz, 1989). Due to the geographic position and topographic situation of the lake, strong winds cause internal waves (seiches) with amplitudes between 2 and 6 m (Horn et al., 1986). Seiches can induce massive displacements of stratified populations (Garneau et al., 2013), thus changing the depth of the spring phytoplankton maximum already on a daily base. Consequently, before sampling we used a fluoroprobe (TS-16-12, bbe Moldaenke GmbH) to determine chlorophyll *a* (Chl *a*) *in situ* profiles between 0 and 30 m. This probe originally distinguishes between four phytoplankton classes (cryptophytes, diatoms, chlorophytes, phycocyanin containing “blue” cyanobacteria) and gives their relative Chl *a* contribution on total Chl *a* concentration (Beutler et al., 2002). We calibrated the probe (optical fingerprint) for the quantification of an additional class, namely the phycoerythrin containing “red” cyanobacterium *Planktothrix rubescens*, which is a dominant primary producer in Lake Zurich (Posch et al., 2012). Based on *in situ* Chl *a* profiles (Figures 1B–D) we determined the current depth with the maximal Chl *a* concentration (see crosses in Figure 1B) and samples (5 L) were taken with a Ruttner water sampler (Uwitec) from this depth layer. These samples were used for the enumeration of bacteria and heterotrophic nanoflagellates (see below), the quantification of algal and ciliate morphotypes (see below), and for chemical analyses. All samples were transported in an insulated box to the laboratory within 30 min. The following chemical parameters were measured: Dissolved phosphorus (DP) with the molybdate method after digestion with H_2_SO_4_ and H_2_O_2_, nitrate (NO_3_) via spectrophotometrical determination after reduction with sodium salicilate Seignette salt, Chl *a* via spectrophotometric measurement after acetone extraction, and dissolved (DOC) and total (TOC) organic carbon via high-temperature catalytic oxidation with a Shimadzu TOC analyzer. We checked the reliability of *in situ* Chl *a* values obtained with the fluoroprobe by comparing values with Chl *a* data determined by extraction (linear regression, *r*^2^ = 0.82).

In addition, profiles of water temperature and oxygen were recorded with a 6600 multi-parameter probe (Yellow Springs Instruments) between 0 and 30 m depth. Profiles of photosynthetically active radiation were determined with a spherical quantum sensor (LI-COR) from the surface in 1-m intervals until an irradiance of < 0.05 μmol quanta m^−2^ s^−1^ was reached.

### Abundance of heterotrophic bacteria and flagellates

Samples for bacterial abundances were fixed with formaldehyde (2% final concentration, f.c.), 1 mL of fixed samples was stained with 10 μL of 4′,6-diamidino-2-phenylindole (DAPI), and total numbers were measured by flow-cytometry (inFlux V-GS, Becton Dickinson). Excitation was set at 355 nm and DAPI emission was measured at 460 ± 50 nm. Further details on the analysis of bacterial parameters (total and filament abundances) are described in Eckert et al. (2012). For the counting of heterotrophic nanoflagellates (HNF), 40 mL of raw water were fixed with Lugol's solution (0.5% f.c.), followed by formaldehyde (2% f.c.), bleached with a few drops of sodiumthiosulfate (3% stock solution), and stored in the dark at 4°C until processing. Fixed samples (5–10 mL) were stained with DAPI, filtered on polycarbonate filter (1-μm pore size) and microscopically counted (*n* = 50–100 flagellates per sample) at 1000 x magnification (Zeiss Axio Imager.M1).

### Abundance of phytoplankton and of ciliate genera and morphospecies

For the determination of algae, 100 mL of water samples were fixed with Lugol's solution (1% f.c.) and analyzed using inverted microscopy (Utermöhl, 1958). Details on phytoplankton species determination and quantification are given in Pomati et al. (2013). Picocyanobacteria, i.e., *Synechococcus*-like coccoid cyanobacteria, were not quantified in this study, as their abundances are very low during spring but start to increase in Lake Zurich at the beginning of July.

For the quantification of ciliate morphotypes we used the Quantitative Protargol Staining (QPS) following the protocol of Skibbe (1994) with few modifications according to Pfister et al. (1999). QPS results in permanent slides and allows for the taxonomic assignments of counted ciliates. Three hundred mL of raw water samples were fixed with Bouin's solution (5% f. c., Skibbe, 1994). Samples were stored at room temperature until further processing. Protargol stained filters (0.8-μm pore sized cellulose nitrate with counting grid, Sartorius) were analyzed microscopically at 1000–1600x magnification. The inspected water volume per sample was at least 9.5 mL, i.e., at minimum 400 ciliates per sample were counted. For ciliate species determination we used the taxonomic key published by Foissner et al. (1999), and we used the higher level taxonomic classification of Lynn (2008). On three selected sampling dates we made triplicate QPS preparations to evaluate standard deviations of ciliate counts. On each sampling occasion, we took also net hauls (mesh size 30 μm) from 10 to 0 m depth for qualitative microscopic observations of living ciliate specimens. These observations gave important background information for the later species determination of fixed specimens on the QPS slides.

### Statistical analyses

For contemporaneous analysis, all collected data were subjected to a Pearson correlation coefficient analysis, performed with the Excel (Microsoft) add-in program XLSTAT-ADA. First parameters were tested for normal distribution and log(x+1) transformed when needed. Pearson correlation coefficients (*r*-values), their signs (positive/negative) and levels of significance (*p*-values) were extracted and exported to the software Cytoscape 3.1.1 for creation of the graphical networks. Additionally, a second graphical network was created using LSA (Ruan et al., 2006) to discover time-shifted associations. We used the eLSA phyton package (http://meta.usc.edu/softs/lsa; Xia et al., 2013) which performs not only a LSA, but also contemporaneous and time-shifted Pearson and Spearman correlation analyses (Xia et al., 2013). A maximal time lag of two steps was set for LSA. As samples were taken in 2–4 day intervals, time lags of two steps range from minimal 4 to maximal 8 days. Finally we compared all statistical approaches concerning the total number and the proportion of shared significant correlations. Further details on the theoretical background and the applicability of networks for community analyses are given by several authors (Ruan et al., 2006; Steele et al., 2011; Fuhrman et al., 2015).

## Results

### Thermal stratification and spring bloom dynamics

A first weak stratification started at the beginning of April and lasted for around 10 days, before being disrupted by a strong wind event (Figures 1A,E). The erosion of thermal stratification was induced by an upwelling internal wave (seiche) of colder hypolimnetic water that led to a sudden cooling of the upper water layer by >3° C within 2 days. During the first stratification, cryptophytes, and diatoms dominated Chl *a* concentrations (Figures 1B,C), and it was the only period when cryptophytes appeared in high numbers. The upwelling seiche also caused a disruption of the first diatom bloom for a few days. Subsequently a second bloom formed in a depth of 6–7 m for around 2 weeks (Figure 1C). From 20 April on, periodic seiches below the surface could be recorded with an amplitude of circa 4 m, as reflected in water temperatures (Figure 1A) but also in spatial concentrations of diatoms (Figure 1C) and *P. rubescens* (Figure 1D).

### Succession phases in lake zurich

Our sampling campaign encompassed four succession phases of plankton dynamics. In the pre-bloom phase (23 March till 3 April) low irradiance and Chl *a* values, and high dissolved phosphorus (DP) concentrations were measured (Figures 1E–G). During this period, *P. rubescens* accounted for 80% of total phytoplankton and ciliates reached a mean abundance of only seven cells mL^−1^ (Figure 1F). As soon as temperature and irradiance increased, we observed a first peak in DOC (Figure 1G) and Chl *a* concentration (mainly due to cryptophytes and diatoms). In parallel, an increase of ciliates (50 cells mL^−1^), heterotrophic bacteria and nanoflagellates (Figure 1H) was recorded. This classical spring bloom situation lasted for 10 days only, resulting in a strong reduction in DP. After this period, a second diatom bloom was also accompanied by high ciliate abundances (60 cells mL^−1^) as well as peaks of bacteria and HNF. Due to increased thermal stratification from 30 April on, *P. rubescens* established a dense metalimnetic population in 10–12 m depth (Figure 1D) and became the dominant primary producer. At this stage, ciliate abundance dropped to about 20 cells mL^−1^. In previous investigations, *P. rubescens* continued to grow in this distinct metalimnetic layer until autumnal mixis caused the erosion of the epi- and metalimnion (Posch et al., 2012).

### Succession of phytoplankton and ciliates

**Figure 2:**
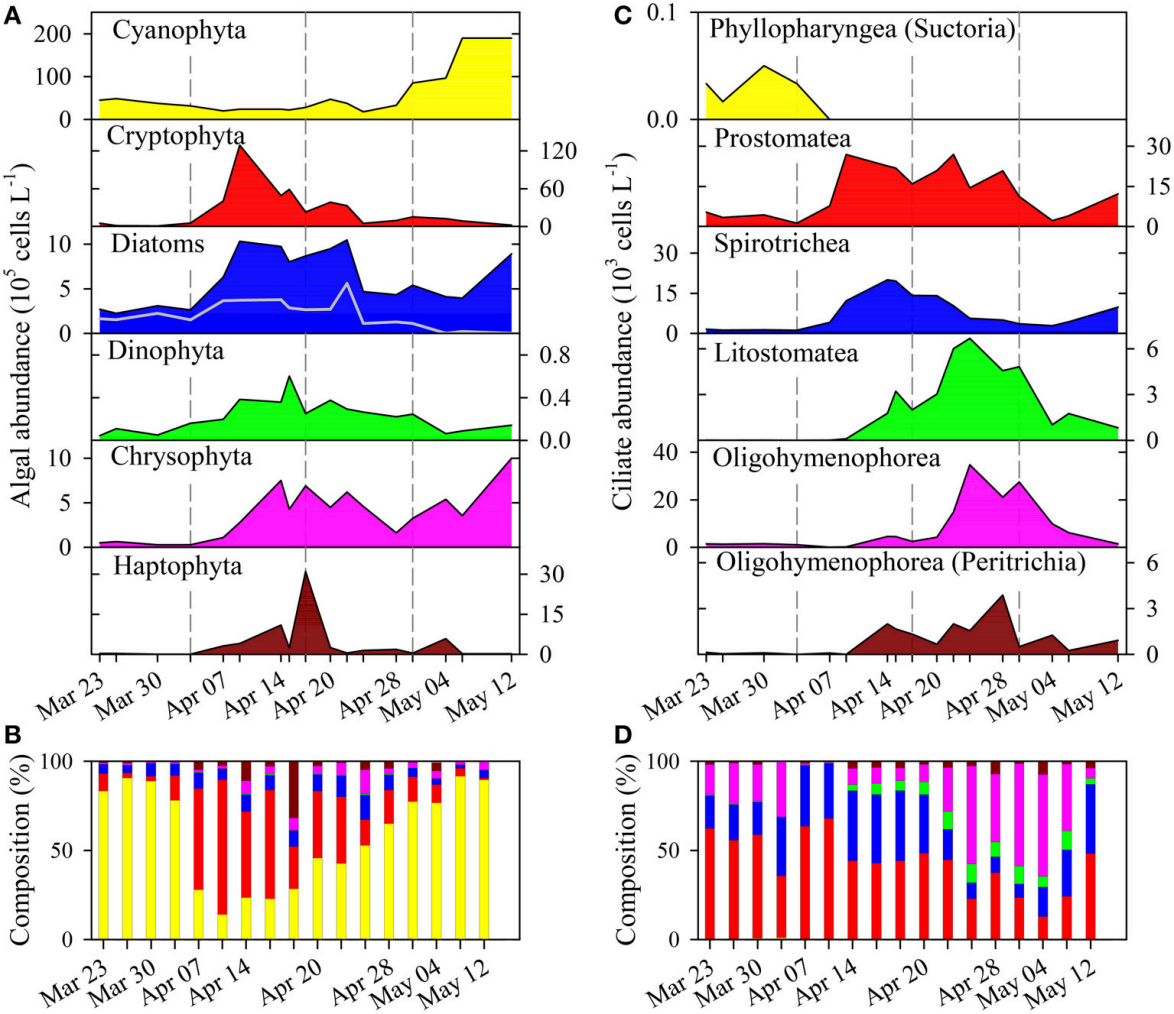
**(A)** Succession of taxonomic phytoplankton groups and **(C)** of ciliate classes based on cell counts. **(B)** Contribution of phytoplankton groups and **(D)** of ciliate groups to total abundance of algae and ciliates, respectively. Note that color code in **(A)** is also valid for **(B)**, and that in **(C)** is also valid for **(D)**. Gray line in the diatoms panel **(A)** show the contribution of colonial diatoms. The class Oligohymenophorea (in **C,D**) is split in two groups: without Peritrichia and Peritrichia only.

**Figure 3:**
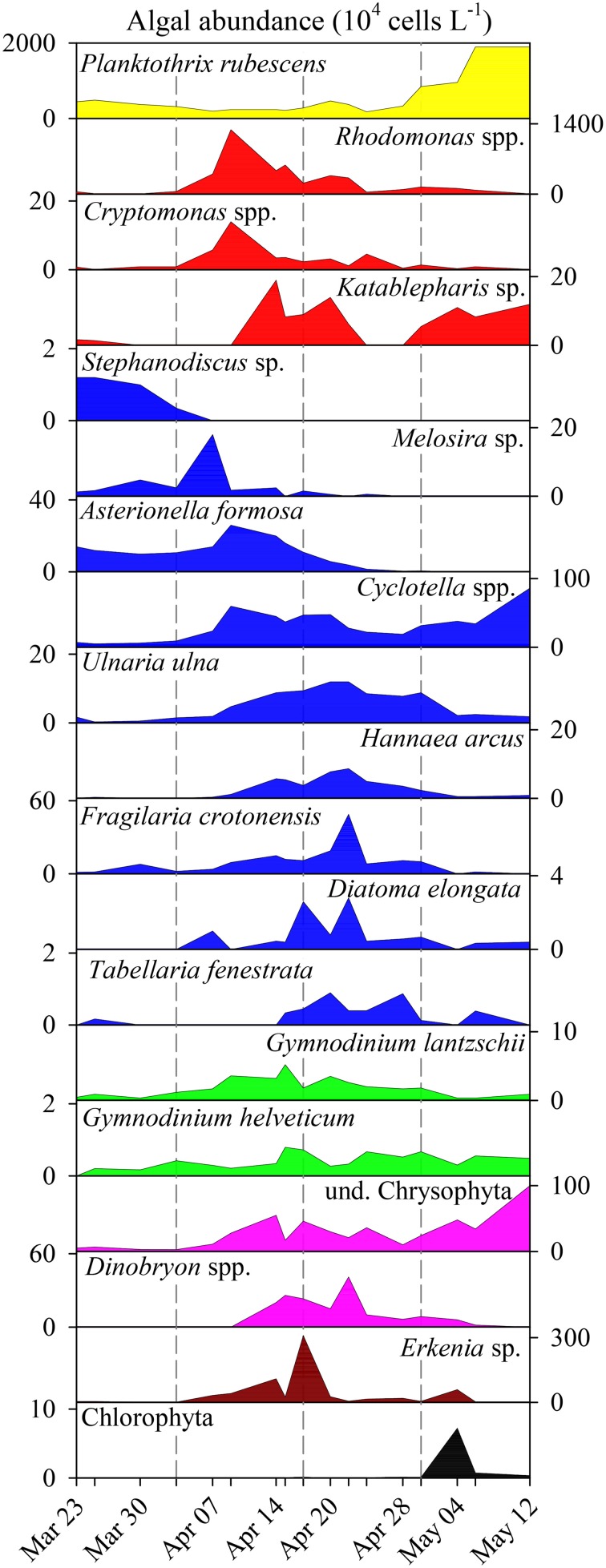
**Abundance (10^4^ L^−1^) of phytoplankton genera/species during spring bloom 2009 in Lake Zurich**. Note the different scaling of y-axes. Genera/species are listed according to their affiliation with taxonomic groups (see color codes in Figure 2A).

The microscopic analysis of phytoplankton showed a clear succession of larger taxonomic groups (Figures 2A,B). For clarity, we present the quantitative data on the 19 determined algal genera/species (Figure 3) that contributed to the composition of larger taxonomic units. The pre-bloom phytoplankton community was dominated by small single celled diatoms but colonial forms were also present, with a few dinoflagellates and the cyanobacterium *P. rubescens*. The classical eukaryotic spring bloom was mainly formed by cryptophytes (*Rhodomonas* spp. and *Cryptomonas* spp.) and single celled diatoms (*Cyclotella* spp.). Afterwards larger sized colonial diatoms (e.g., *Tabellaria fenestrata, Fragilaria crotonensis*; Figure 3) followed, accompanied by dinoflagellates (*Gymnodinium* spp.) and chrysophytes (*Dinobryon* spp.). *P. rubescens* dominated the fourth successional phase. Throughout the entire succession of population waves, we observed continuous high abundances of single celled diatoms (*Cyclotella* sp.) and of undetermined chrysophytes.

**Figure 4:**
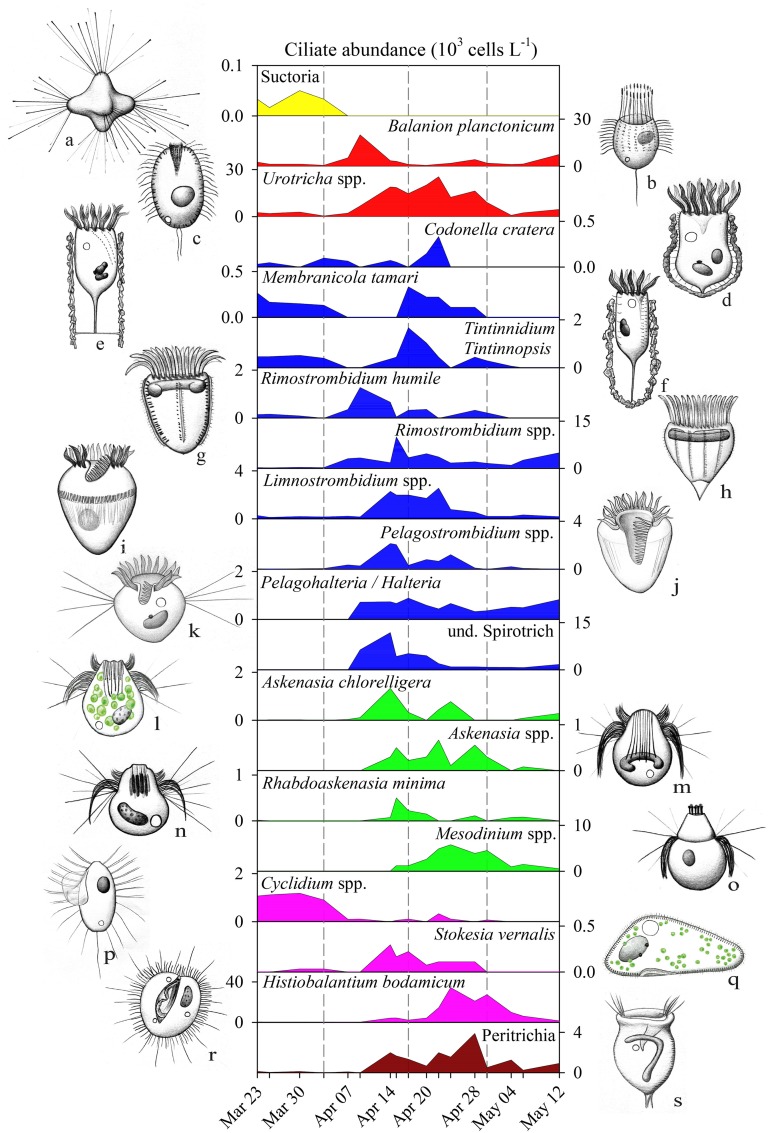
**Abundance (10^3^ L^−1^) of ciliate genera/species during spring bloom 2009 in Lake Zurich**. Note the different scaling of y-axes. Ciliate genera/species are listed according to their affiliation with taxonomic groups (see also Table 1 and color codes in Figure 2C). Drawings of ciliates do not show the right size proportions of specimens to each other. (a) *Staurophrya elegans*, (b) *Balanion planctonicum*, (c) *Urotricha* spp., (d) *Codonella cratera*, (e) *Membranicola tamari*, (f) Tintinnids, (g) *Rimostrombidium humile*, (h) *Rimostrombidium* spp., (i), *Limnostrombidium* spp., (j) *Pelagostrombidium* spp., (k) *Pelagohalteria*/*Halteria*, (l) *Askenasia chlorelligera*, (m) *Askenasia* spp., (n) *Rhabdoaskenasia minima*, (o) *Mesodinium* spp., (p) *Cyclidium* spp., (q) *Stokesia vernalis*, (r) *Histiobalantium bodamicum*, (s) Peritrichia. Drawings are original artworks by G. Pitsch.

Ciliate classes also showed a clear temporal succession (Figures 2C,D). Details on the dynamics of prominent species or genera are given in Figure 4 and Table 1. The pre-bloom phase was the only time when Suctoria (class Phyllopharyngea, *Staurophrya elegans*) and small scuticociliates (class Oligohymenophorea, *Cyclidium* spp.) were found in higher numbers. Lorica bearing ciliates of the class Spirotrichea (*Codonella cratera, Membranicola tamari, Tintinnidium* sp., *Tintinnopsis* sp.) were also detected. As soon as cryptophytes and diatoms increased (bloom phase), *Balanion planctonicum* (Prostomatea) and *Rimostrombidium humile* (Spirotrichea, Choreotrichida) showed steep increases in numbers (Figure 4). Colonial diatoms were often colonized by peritrichous ciliates (Oligohymenophorea), and algivorous as well as mixotrophic Litostomatea (e.g., *Askenasia* spp.) increased. During the post bloom phase ciliates of the class Prostomatea were still abundant, however, *B. planctonicum* was replaced by various *Urotricha* species (Figure 4). During this period a single scuticociliate *Histiobalantium bodamicum* (class Oligohymenophorea) formed up to 35 cells mL^−1^ (i.e., 55% of total abundance), accompanied by a highly abundant genus of the class Litostomatea, namely *Mesodinium* sp.. Based on abundance data, groups contributed to the total ciliate assemblage in the following order (Figure 2D): Prostomatea (43.3%), Oligohymenophorea (26.4%), Spirotrichea (25.3%), Litostomatea (4.9%), and Phyllopharyngea (0.1%).

**Table 1:** **Ciliate species/genera detected during the phytoplankton spring bloom period of Lake Zurich in 2009**.

	**Frequency: % Average (Maximum)**	**Abundance: ciliates L^−1^ Average (Maximum)**
**PHYLLOPHARYNGEA**		
Suctoria		
Endogenida		
*Staurophrya elegans*	0.1 (0.9)	8 (50)
**PROSTOMATEA**		
Prorodontida		
*Balanion planctonicum*	16.0 (50.6)	3556 (19982)
*Urotricha* spp.	27.5 (47.0)	9483 (25492)
**SPIROTRICHEA**		
Tintinnida		
*Codonella cratera*	0.3 (2.8)	51 (334)
*Tintinnidium*/*Tintinnopsis*	2.5 (11.3)	391 (1670)
*Tintinnidium pusillum*		
*Tintinnopsis cylindrata*		
*Membranicola tamari*	0.8 (3.8)	101 (334)
Choreotrichida		
*Rimostrombidium humile*	1.0 (3.2)	245 (1280)
*Rimostrombidium* spp.	9.3 (25.7)	2690 (10064)
*Rimostrombidium hyalinum/brachykinetum*		
*Rimostrombidium lacustris*		
Strombidiida		
*Limnostrombidium* spp.	2.6 (5.5)	827 (2560)
*Limnostrombidium pelagicum*		
*Limnostrombidium viride*		
*Pelagostrombidium* spp.	1.4 (4.2)	499 (2152)
*Pelagostrombidium fallax*		
*Pelagostrombidium mirabile*		
Sporadotrichida		
*Halteria*/*Pelagohalteria*	1.3 (3.6)	431 (891)
*Pelagohalteria cirrifera*		
*Pelagohalteria viridis*		
*Halteria* sp.		
Undetermined Spirotrich	5.7 (23.3)	2385 (11874)
**LITOSTOMATEA**		
Cyclotrichiida		
*Askenasia chlorelligera*	0.6 (2.6)	261 (1336)
*Askenasia* spp.	0.4 (1.1)	178 (668)
*Askenasia acrostomia*		
*Rhabdoaskenasia minima*	0.2 (1.0)	72 (500)
*Mesodinium* sp.	3.7 (9.5)	1605 (5789)
**OLIGOHYMENOPHOREA**		
Peritrichia		
Sessilida	2.6 (7.3)	970 (3896)
*Vaginicola* sp.		
*Vorticella natans*		
*Vorticella vernalis*		
Peniculia		
Peniculida		
*Stokesia vernalis*	0.2 (1.0)	68 (297)
Scuticociliata		
Pleuronematida		
*Histiobalantium bodamicum*	19.3 (57.5)	7741 (34509)
*Cyclidium* spp.	4.4 (25.5)	304 (1186)
**Rare species**		*Actinobolina smalli* (Litostomatea)
*Coleps spetai* (Prostomatea)		*Belonophrya pelagica* (Litostomatea)
*Epistylis anastatica* (Oligohymenophorea)		*Monodinium armatum* (Litostomatea)
*Epistylis pygmaeum* (Oligohymenophorea)		*Lagynophrya acuminata* (Litostomatea)
*Epicarchesium pectinatum* (Oligohymenophorea)		*Pelagodileptus trachelioides* (Litostomatea)
*Cinetochilum margaritaceum* (Oligohymenophorea)		*Pelagovasicola cinctum* (Litostomatea)

*The 20 clearly definable ciliate morphotype units were identical to described species, or comprised two or more species within a genus. Eleven clearly recognizable but rare species were excluded from graphical presentation and statistical analyses, as these species were found in too low numbers or only on single sampling dates*.

### Diversity of pelagic ciliates

In total we could quantify the succession of 20 clearly definable ciliate morphotype units (Figure 4). In many cases these morphotypes were identical to described species (Table 1), while others comprised two or more species from one genus (e.g., *Limnostrombidium, Urotricha*) or from an even larger taxonomic group (Sessilida) which could not be further identified to species level. Specifically, the quantification of peritrichous ciliates, often epibionts on colonial diatoms or crustaceans, is not possible solely by QPS due to restrictions in the filterable water volume (i.e., to concentrate sufficient colonial diatoms and crustaceans). By taking additional net-hauls we could nevertheless determine some peritrichous taxa to the species level (Table 1). Finally, 11 clearly recognizable but rare species (Table 1) were excluded from the statistical analyses, as these species were found in too low numbers (<0.2 cells mL^−1^) or only on single sampling dates. These rare species nevertheless formed 34% of total ciliate richness (31 taxa) observed within a rather short investigation period of 7 weeks.

### Species-specific associations during the spring bloom

**Figure 5:**
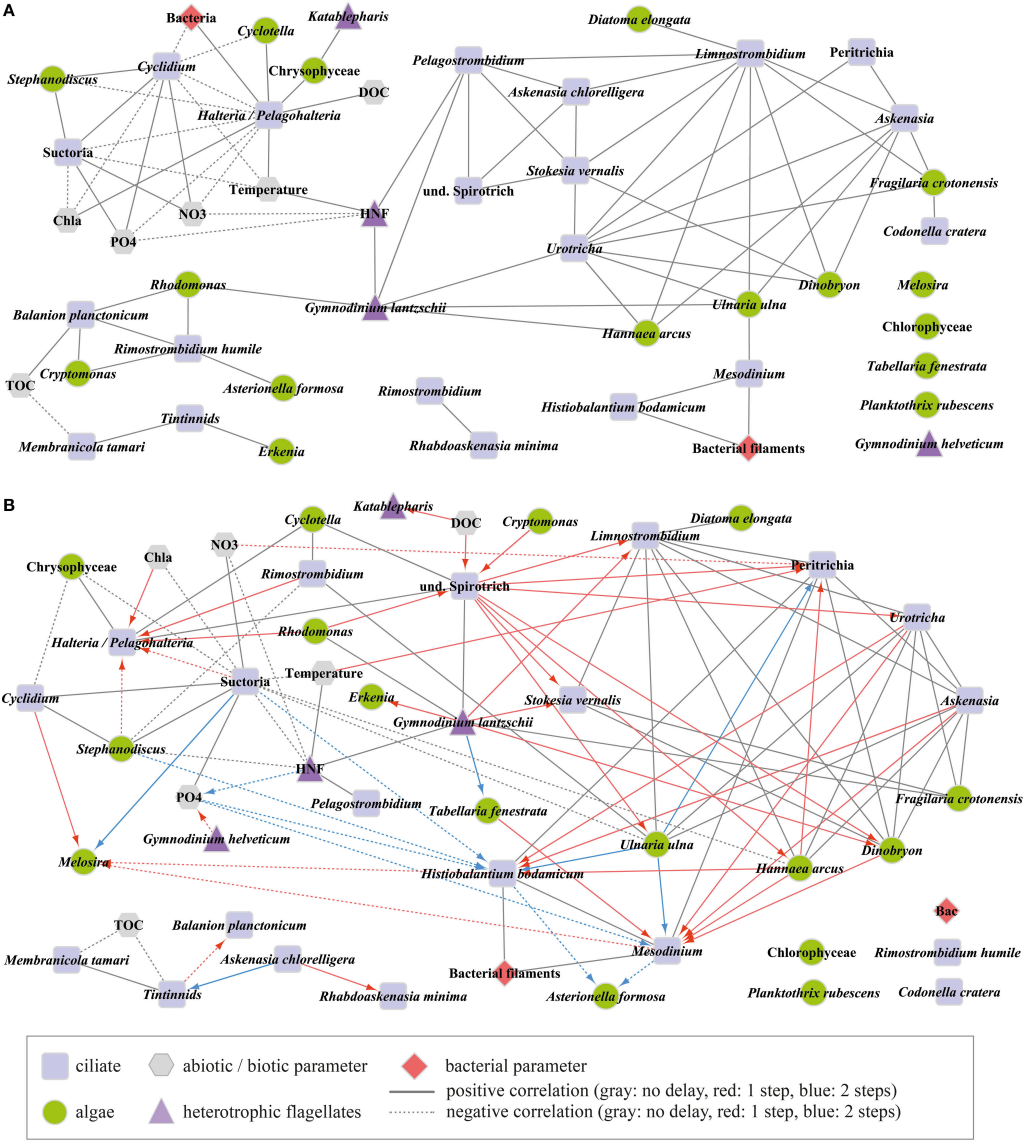
**Microbial community network diagram based on (A) Pearson correlation analysis and (B) time-shifted local similarity analysis**. Ciliate species/genera, heterotrophic nanoflagellates (HNF), and large heterotrophic flagellates were set as central nodes. Connections (edges) show significant connections (*p* ≤ 0.003). Different colors in **(B)** show contemporaneous and time-shifted associations. Arrows point to the parameter that was delayed. Central nodes without any significant association to any parameter are shown at the bottom right. Abbreviations: Chla, total chlorophyll a; DOC, dissolved organic carbon; NO3, nitrate; PO4, dissolved phosphorus; TOC, total organic carbon.

Networks (Figure 5) show parameters (termed as nodes) and correlations/associations between nodes as lines (termed as edges, different line-styles show positive and negative connections, respectively). For LSA, different colors of edges indicate time-shifted associations in our networks. Ciliate species/genera, heterotrophic nanoflagellates (HNF) and larger heterotrophic flagellates were set as central nodes, i.e., all significant correlations/associations (*p* ≤ 0.003) between these parameters are depicted. Connections of central nodes with the remaining nodes (phytoplankton species, other biotic, and abiotic parameters) are also shown, but for clarity not the connections between the remaining nodes.

**Figure 6:**
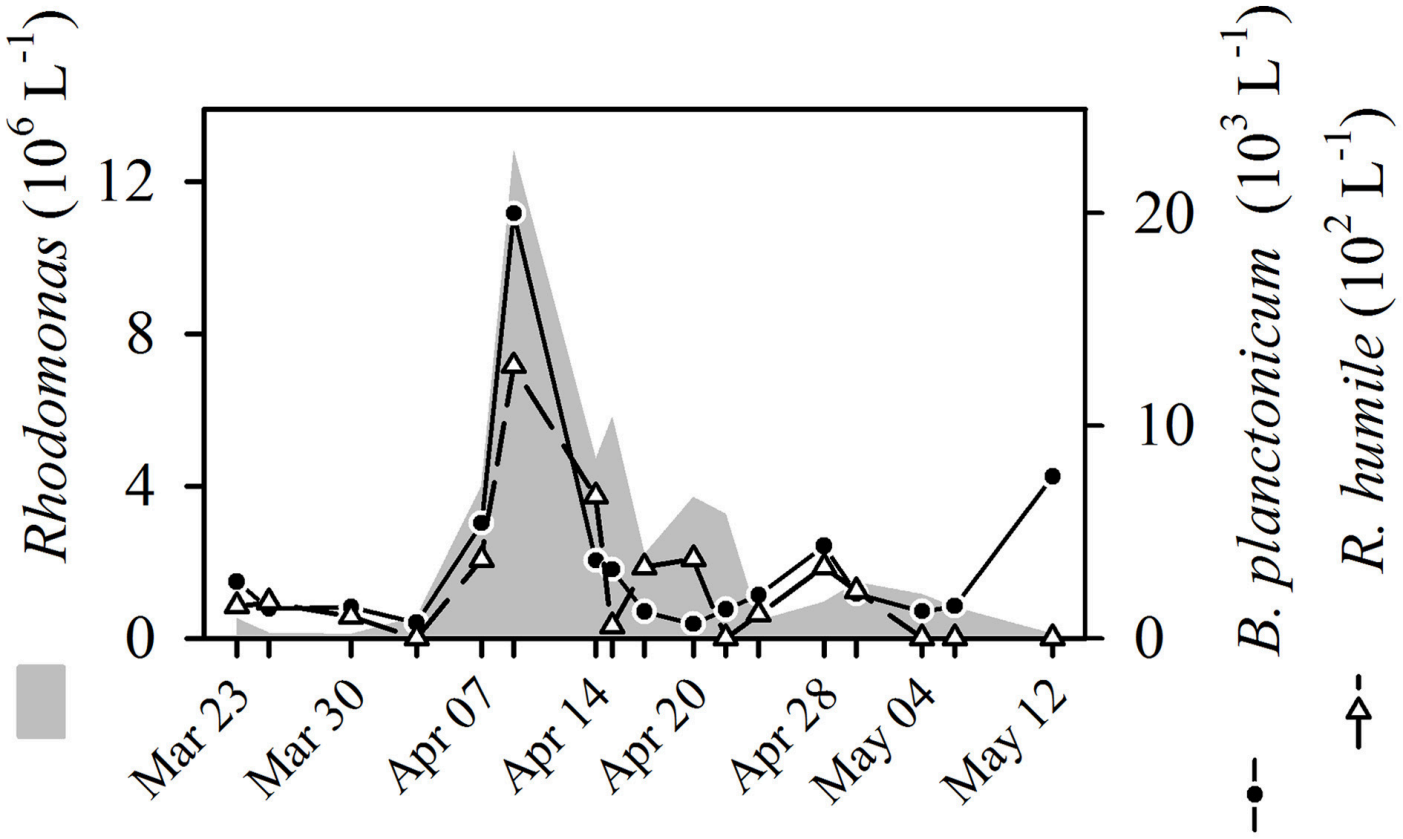
**Dynamics of the cryptophyte ***Rhodomonas*** sp. and the ciliates ***Balanionplanctonicum*** and ***Rimostrombidium humile*** during spring bloom 2009 in Lake Zurich**.

The contemporaneous Pearson correlation analysis (PCC, Figure 5A) resulted in 116 significant (*p* ≤ 0.003) pairs, i.e., 89 positive and only 27 negative correlations out of 1225 possible combinations (Table 2). Through the selection of central nodes, 66 positive and 14 negative correlations are shown. They are depicted in one network comprised of one larger cluster (10 connected central nodes) and several smaller clusters (2–3 connected central nodes). *Rimostrombidium* and *Rhabdoaskenasia minima* formed an independent cluster. Abundances of *Gymnodinium helveticum* and four algal morphotypes did not correlate with any other parameter. The algae *Rhodomonas lens* and *Cryptomonas* spp. were linked by only positive correlations with the algivorous ciliates *B. planctonicum* and *R. humile* (see also Figure 6), together with the dinoflagellate *Gymnodinium lantzschii*. A group of mixotrophic/omnivorous ciliate species in our network was formed by *Askenasia chlorelligera* and *Stokesia vernalis*, two species containing endosymbiotic green algae (zoochlorellae, Stoecker et al., 2009). In addition, two other kleptoplastidic ciliate species were in this group, i.e., *Limnostrombidium viride* and *Pelagostrombidium mirabile*, which only temporarily retain the chloroplasts of ingested algae (Rogerson et al., 1989). The morphotype unit *Halteria*/*Pelagohalteria* (Table 1) showed the only significant positive correlation with the abundances of heterotrophic bacteria (Figure 5A). The filter feeders *Cyclidium* spp. (*C. glaucoma* and unidentified species) were negatively correlated with heterotrophic bacteria. A second cluster of ciliates related to the dynamics of filamentous bacteria was formed by *Mesodinium* sp. and *H. bodamicum* (Figure 5A). Three ciliate genera (*Limnostrombidium* spp., *Askenasia* spp. and *Urotricha* spp.) were all positively related to each other, but also to colonial diatoms (*F. crotonensis, Hannaea arcus*), a single-celled diatom (*Ulnaria ulna*), and the colonial mixotrophic chrysophyte *Dinobryon* spp. (Figure 5A).

**Table 2:** **Comparison of significant (***p*** ≤ 0.003) correlations/associations out of 1225 possible pairs detected by different contemporaneous and time-shifted (1, 2 steps) analyses**.

	**Pearson correlation**	**Spearman correlation**	**Local similarity analysis**	**Pearson correlation time-shifted**	**Spearman correlation time-shifted**
Contemporaneous	116 (89/27)	148 (105/43)	90 (66/24)	90 (73/17)	109 (79/30)
1 step	–	–	51 (39/12)	100 (91/9)	137 (104/33)
2 steps	–	–	24 (17/7)	58 (56/2)	99 (73/26)
Total	116 (89/27)	148 (105/43)	165 (122/43)	248 (220/28)	345 (256/89)

*Number in brackets show positive/negative correlations*.

**Figure 7:**
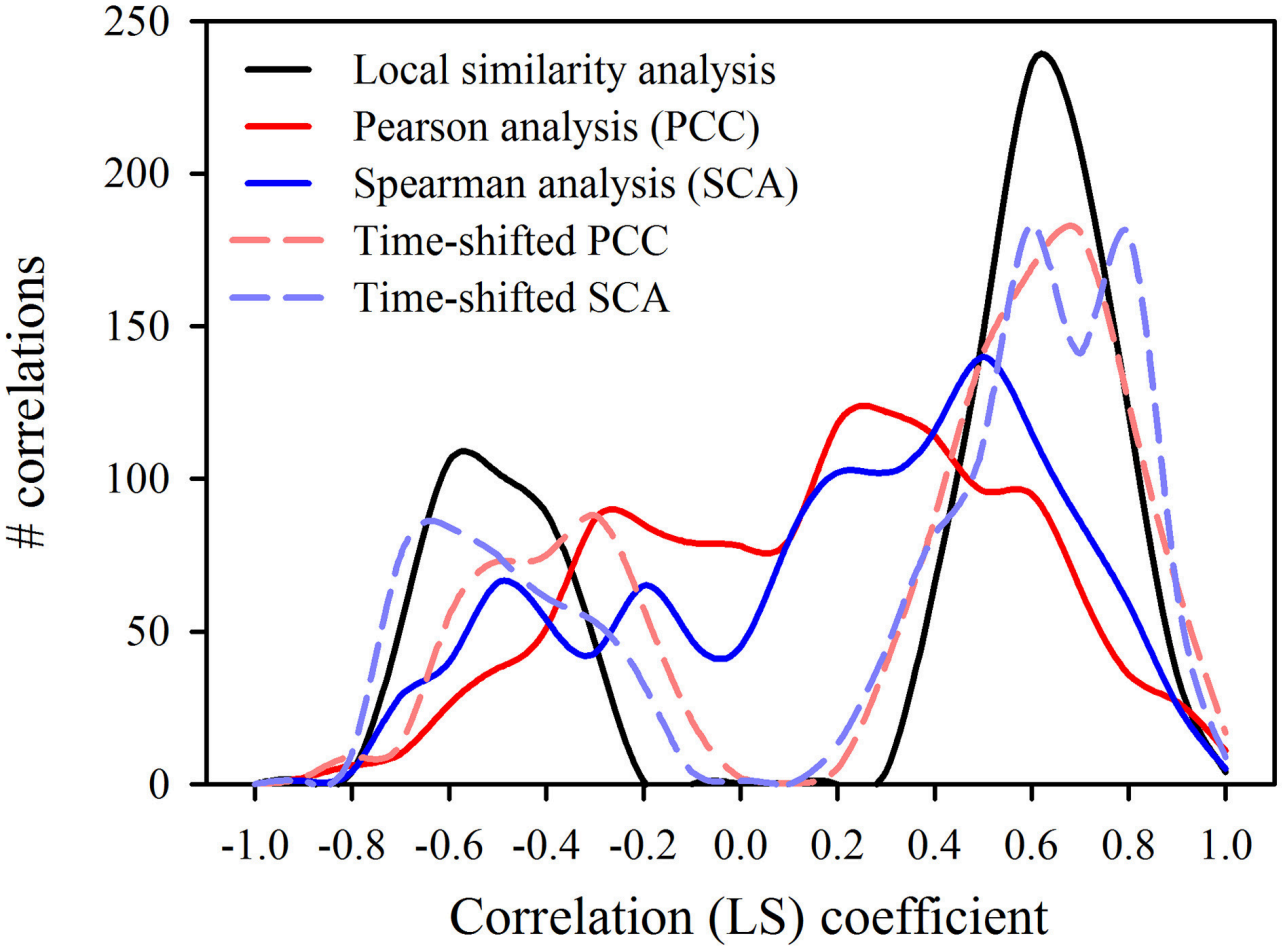
**Distribution of correlation coefficients (LS) calculated for 1225 possible pairs by different contemporaneous and time-shifted analyses**.

The time-shifted LSA (Figure 5B) gave 165 significant (*p* ≤ 0.003) pairs, i.e., 122 positive and 43 negative connections (Table 2). The majority of significant associations was contemporaneous (90 pairs), followed by 51 cases with a time shift of one step and only 24 cases with a delay of two steps (Table 2). For the depiction of the network we selected only correlations between central nodes and those of central nodes with other factors (algae and abiotic parameters). This reduced the number of presented correlations to 56 (contemporaneous), 37 (one step delay), and 13 (two steps delay), respectively. The network based on LSA shows one large cluster with 15 connected central nodes, and one smaller group of 5 connected ciliates. The two ciliates *R. humile* and *C. cratera*, two algal species and heterotrophic bacteria were not associated with any other parameter. Some connections within ciliates and also of ciliates with algae were found by both, PCC and LSA, e.g., the association of *Halteria*/*Pelagohalteria* with Chrysophyceae and *Cyclotella*. A striking difference was detected for ciliates connected with *Rhodomonas*. In contrast to the results from PCC, this algal genus showed a one-step time-shifted association with *Halteria*/*Pelagohalteria* and an undetermined spirotrichous ciliate but no connections to *B. planctonicum* and *R. humile*. However, by comparing all significant associations (*p* = 0.003) detected by both, PCC and LSA, we found 66 shared pairs (Table 3). Time-shifted Pearson and Spearman correlation analyses resulted in much higher numbers of total and shared significant correlations (Tables 2, 3) but were not further used for the creations of networks. Figure 7 shows the effects of LSA and the two other time shifted analyses on the distribution of correlations factors. All time shifted analyses caused a distinctive shift of correlation factors toward > 0.2 or < −0.2 (Figure 7).

**Table 3:** **Comparison of shared significant (***p*** ≤ 0.003) correlations between different contemporaneous and time-shifted analyses**.

	**Pearson correlation**	**Spearman correlation**	**Local similarity analysis**	**Pearson correlation time-shifted**	**Spearman correlation time-shifted**
Pearson correlation	**116**	–	–	–	–
Spearman correlation	73	**148**	–	–	–
Local similarity analysis	66	103	**165**	–	–
Pearson correlation time-shifted	113	86	110	**248**	
Spearman correlation time-shifted	83	148	158	161	**345**

*Bold values are total significant correlations per analysis*.

## Discussion

### Cryptophytes and their predators

Several field surveys have shown that *B. planctonicum* was the first and most effective grazer of cryptophytes in spring (Müller, 1991; Sommaruga and Psenner, 1993; Šimek et al., 2014). A 12 years data analysis showed that this predator-prey relationship was a predictable phenomenon in Lake Constance throughout the whole investigation period (Tirok and Gaedke, 2007). Numerical and functional response curves of *B. planctonicum* isolates demonstrated that it is a typical r-strategist. In addition, this species reached maximal growth rates at lower temperatures than competing ciliates (Müller and Schlegel, 1999). Furthermore, there is a niche separation to other small sized prostomatid ciliates, namely *Urotricha* spp. (Weisse et al., 2001), which can feed on similar sized but other types of prey. This observation was also reflected in our networks (Figure 5) by the lack of direct links between *B. planctonicum* and *Urotricha* spp.

Only by PCC, we found strong positive correlations of the filter feeding ciliate *R. humile* with cryptophytes (Figures 5A, 6). The congener *R. lacustris* is known as efficient consumer of cryptophytes in spring (Müller and Schlegel, 1999). However, this has not been reported for *R. humile* so far, although this species was found in higher population densities than *R. lacustris* in many lakes (Pfister et al., 2002; Sonntag et al., 2006). Our results, moreover, are in accordance with laboratory studies (Müller and Schlegel, 1999) showing that the diatom *Stephanodiscus* was not a suitable food source for either *B. planctonicum* or for *Rimostrombidium* spp., as no significant relations between these organisms could be detected. The colorless dinoflagellate *G. lantzschii* was the third protistan species apparently profiting from cryptophytes as food source. This co-occurrence (Figure 5) is described as predator prey relationship (Weisse and Müller, 1998). We regularly detected *Gymnodinium* cells with ingested cryptophytes in our Protargol preparations (data not shown). In fact, these dinoflagellates seem to be voracious omnivores, ingesting even prey of their own cell size. Besides cryptophytes, we also found ingested ciliates and centric diatoms in fixed and living *Gymnodinium* cells.

### Association of mixotrophic/omnivorous ciliates

The co-occurrence of four ciliate genera/species (*A. chlorelligera, S. vernalis, Limnostrombidium* spp, *Pelagostrombidium* spp., Figures 4, 5A) reflects observations from oligo- and mesotrophic lakes that mixotrophic/omnivorous species followed the first peak of ciliates in spring (Amblard et al., 1993; Sonntag et al., 2006; Stoecker et al., 2009). However, this species association was only obvious from the network based on PCC but not from LSA. The triggers for the rise of ciliates with zoochlorellae/kleptoplastids are not known. Mixotrophic ciliates are competitors of strictly heterotrophic species when algal prey is rare (see references in Stoecker et al., 2009). Additionally, successful feeding is also linked to prey accessibility (size and shape of algae). In our study we observed a modest increase of colonial diatoms in parallel with the appearance of mixotrophic/omnivorous ciliates (see Figures 3, 4), possibly limiting the spectrum of available food for ciliates. An alternative hypothesis for the appearance of mixotrophs was presented by Sonntag et al. (2011): some *Chlorella* bearing ciliates were more resistant to solar ultraviolet radiation than heterotrophic ones, thus allowing for a niche partitioning between these two lifestyles.

### Bacterivorous ciliates

*Halteria* is known as a quantitatively important bacterivore, sometimes dominating total protistan grazing rates, and thus even exceeding the grazing impact of HNF (Šimek et al., 2000). Nevertheless, only PCC but not LSA revealed a correlation between *Halteria* and bacteria (Figures 5A,B). The strong positive associations of this taxonomic group with the centric diatom *Cyclotella* probably mirrors a predator-prey relation (Skogstad et al., 1987). Abundant small sized (4–6 μm) centric *Cyclotella* (cf. *melosiroides*) in Lake Zurich were within the preferred prey size range of *Halteria* (Jürgens and Šimek, 2000), thus serving as a potential second food source.

We have to interpret the negative correlation (only detected with PCC) of *Cyclidium* spp. with bacterial abundance with care, and we suppose that it reflects non-overlapping seasonal peaks and not necessarily a direct causal link. *Cyclidium* spp. typically reach highest abundances during the cold seasons in the upper water strata in meso- to oligotrophic lakes, and they dominate the cold hypolimnion during the rest of the year (Sonntag et al., 2006). Both habitats are characterized by low bacterial abundances and production, which contradicts the high half saturation constants for bacterial prey (Posch et al., 2001). Probably, the decline of *Cyclidium* spp. is linked to competition with other bacterivores or shifts in the bacterial assemblage (Eckert et al., 2012; see also Figure 2 in Salcher, 2014).

Both, PCC and LSA, might indicate that *Mesodinium* sp. and *H. bodamicum* fed on filamentous bacteria (Figures 5A,B). Their size range of ingestible prey is large, also feeding on algae, flagellates and small ciliates (Müller and Weisse, 1994; Foissner et al., 1999). The preference for large prey particles might explain why we found correlations of the two species only with filamentous bacteria, but not with total heterotrophic bacteria which are dominated by tiny coccoid and rod-shaped morphotypes (Salcher, 2014).

### Unresolved co-occurrence patterns of ciliate and algal species

We found numerous contemporaneous and time-shifted associations of *Urotricha* spp., *Askenasia* spp. and *Limnostrombidium* spp. with colonial diatoms and chrysophytes (Figure 5). These colonial algae are too large for being ingested by *Urotricha* spp. Solitary *Fragilaria* and *Dinobryon* cells were occasionally present but in too low numbers to sustain these ciliate populations. In addition, the single celled diatom *Ulnaria ulna* is not an appropriate food source for these ciliates due to its large cell size (up to 350 μm). The morphotype unit *Urotricha* spp. included various species, and QPS preparations did not allow for detailed taxonomic determinations. The various associations to other organisms probably reflects that too many different species were pooled in this morphotype unit. For a proper identification of the so far 13 described euplanktonic *Urotricha* species, the use of silver carbonate was recommended (Foissner and Pfister, 1997) in combination with live observations. We hope that in the future 18S rDNA sequencing might allow for a detailed species determination, as *Urotricha* species greatly differ in their preferred food sources but also prey size ranges.

### Interpretation of contemporaneous and time shifted correlations

LSA was originally developed to detect spatial or time shifted associations which are not discovered by contemporaneous analyses alone (Ruan et al., 2006). Beside the analysis of environmental sequencing data, LSA can be also applied on classical counting data as stated by the authors (Ruan et al., 2006). Since then, it is discussed how to interpret these association patterns, and how to detect possible causalities (Faust and Raes, 2012; Fuhrman et al., 2015). It was supposed that positive associations indicate mutualism, commensalism, and cross-feeding activities. Negative associations may point to parasitism, predation, and competition. Nevertheless, the probability that correlations indeed mirror these theoretical assumptions, are influenced, e.g., by the generation time of involved organisms, the turnover time of available nutrients, and also the sampling resolution at which microbial dynamics are observed. Finally, a predator-prey relation between two organisms might be influenced by a third factor (e.g., bottom-up control through nutrients of the prey, top-down control of the predator).

Our data set offers a striking example: We found a clear positive contemporaneous correlation of *Rhodomonas* with the raptorial feeder *B. planctonicum* (Figures 5A, 6), which is a definite predator-prey relationship (see details above). The growth of *Rhodomonas* is linked to high phosphorus concentrations, increased insolation and stable thermal stratification in spring. Ciliates can reach equivalent growth rates as algae, thus, abundances of prey and predators coincided. Finally, we even observed a synchronous decline of *Rhodomonas* and *B. planctonicum*, possibly caused by physical forces of an internal wave or by phosphorus limitation of cryptophytes. Additionally, the abundance of metazooplankton (rotifers, daphnids) increased (data not shown), exerting a top-down control on both, algae and ciliates. This example highlights that predator-prey interactions of protists may be indeed positively correlated when the sampling effort is high enough to follow the dynamics at high temporal resolution. However, we have to state that the co-occurrence between *Rhodomonas* and *B. planctonicum* was only reflected by PCC and not by LSA.

Although LSA proved to be successful in finding time-shifted patterns in several studies (Needham et al., 2013; Chow et al., 2014), this statistical approach was not adequate for the evaluation of our data describing population dynamics at a high temporal resolution. The fast succession of single and not repetitive short-living population peaks of various protists, which is a main character of spring bloom dynamics in freshwater, caused an over-proportional high number of significant connections when analyzed with LSA (Table 2, Figure 7). Nevertheless, network based analyses may help to formulate testable hypotheses about possible interactions of species that co-occur, co-vary or do not co-occur (Chow et al., 2014). However, to verify these hypotheses, it is still necessary to recognize and isolate protists for further experiments.

## Author contributions

TP, JP, FP designed research. BE performed ciliate analyses. GP performed ciliate drawings. EE performed bacterial analyses, FP algal analyses. TP analyzed data and created figures. TP and BE wrote the publication.

### Conflict of interest statement

The authors declare that the research was conducted in the absence of any commercial or financial relationships that could be construed as a potential conflict of interest.
